# Effect of Displacement on Adherence to TB Treatment: An Observational Study in TB Patients from Internally Displaced Persons of Pakistan

**DOI:** 10.12669/pjms.37.3.2992

**Published:** 2021

**Authors:** Farman Ullah Khan, Zahid Asghar, Muhammad Khalid Tipu, Asim.ur Rehman, Asif Khan, Tofeeq Ur-Rehman

**Affiliations:** 1 Farman Ullah Khan, Department of Pharmacy, Faculty of Biological Science, Quaid-i-Azam University, 45320 Islamabad, Pakistan; 2 Zahid Asghar, School of Economics, Faculty of Social Sciences, Quaid-i-Azam University, 45320 Islamabad, Pakistan; 3 Muhammad Khalid Tipu, Department of Pharmacy, Faculty of Biological Science, Quaid-i-Azam University, 45320 Islamabad, Pakistan; 4 Asim.ur.Rehman, Department of Pharmacy, Faculty of Biological Science, Quaid-i-Azam University, 45320 Islamabad, Pakistan; 5 Asif Khan, District TB Control Program, Bannu, 28100 Khyber PakhtunKhwa, Pakistan; 6 Tofeeq Ur-Rehman, Department of Pharmacy, Faculty of Biological Science, Quaid-i-Azam University, 45320 Islamabad, Pakistan

**Keywords:** Tuberculosis, Displacement, Internally Displaced Persons, Adherence, TB Treatment Outcomes

## Abstract

**Objective:**

This study was aimed to investigate TB patients adherence and treatment outcomes among internally displaced patients in comparison with adjacent settled areas.

**Methods:**

The study was designed as an observational cross-sectional study among the TB patients of internally displaced populations (IDPs) of North Waziristan Agency (NWA) and adjacent settled areas of Bannu and Lakki Marwat (NIDPs). Based on the study inclusion and exclusion criteria 330 patients fullfilled the inclusion criteria and were assigned equally to both IDPs and NIDPs study groups. Odds ratio (OR) with 95% confidence interval was calculated and p-values, 0.05 were considered statistically significant.

**Results:**

The treatment outcomes with the status of “cured” and “completed treatment” were better among NIDPs as compared to IDPs. Patients with treatment outcome status of “defaulted treatment”, “without documentary evidence, and “failure” were high in IDPs as compared to NIDPs. Adherence to TB treatment was better among NIDPs (50.9%) as compared to IDPs (39.4%). The patients showing non-adherence to TB treatment were more among IDPS (27.3%) than NIDPs (10.9%).

**Conclusion:**

Overall results of this study revealed a poor adherence to the TB treatment medications with an odds ratio of 0.286, (p<0.05) among IDPs as compared with NIDPs.

## INTRODUCTION

Tuberculosis (TB) is a bacterial infection caused by mycobacteria, residing in human. The pathogenic infection kills 1.7 million people every year in the world particularly in Asia.^1^,^2^ Pakistan ranks 5^th^ in the world among high TB burden countries and shares 61% of the burden in the WHO Eastern Mediterranean Region.^2^,^3^ The goal of TB treatment is to make an individual free of the disease and to reduce the chance of spread of disease. Treatment of TB requires multiple medications daily for months, for the eradication of infection which largely depends upon adherence to treatment protocols. The adherence to the treatment protocol is a critical challenge faced by most of the TB control programs of developing countries.^4^

A patient with lower income, living in over-crowded and poor hygiene condition is more likely to show non-adherence with the therapy.^5^ TB patients, who do not show adherence to the treatment protocols, are not only at risk of relapse but also contribute to further transmission and development of resistance.^4^ Every year more than 400,000 worldwide and in Pakistan around 15000 new multidrug resistance TB cases are reported.^6^ TB is again emerging as a global health issue with increased prevalence among peoples living in poor hygiene conditions.^7^ United Nations High Commissioner for Refugees recorded a score of 42.5 million forcibly displacements globally during 2015 which has forced the people to live in compromised hygienic conditions and medical facilities.^8^,^9^

In addition to 1.54 million refugee, Pakistan had to take care of a large number of internal displacements, due to natural disaster and internal armed conflict of 2014.^10^ The counter militant operations “Zarb-e-Azb” was launched on 15^th^ June 2014 in the North Waziristan Agency (NWA), a Federally Administered Tribal Area (FATA) of Pakistan, and resultantly a large portion of the residents was forced to move to the adjoining areas like Lakki Marwat, Bannu, Frontier region of Bannu (F.R. Bannu) and Dera Ismail Khan (D.I. Khan). United Nation Office has registered 961,000 internally displaced persons from NWA, who were forced to live in compromised hygienic conditions and medical facilities posing a challenge in TB control.^11^ This study was aimed to investigate adherence and treatment outcomes among TB patients of internally displaced populations (IDPs) of North Waziristan Agency (NWA) and adjacent settled areas of Bannu and Lakki Marwat.

## METHODS

**Fig.1:**
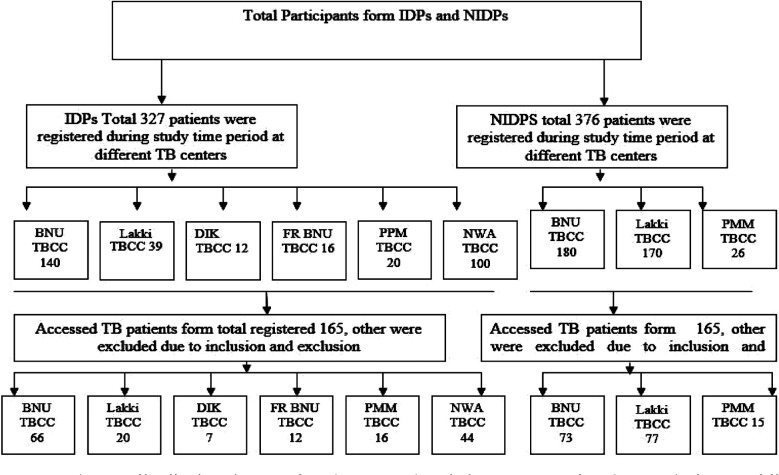
IDPs (Internally displaced TB patients), NIDPs (Settled areas TB patients) PPM (Private Public Mix) F.R Bannu (Frontier Region Bannu), NWA (North Waziristan agency), DI Khan (Dera Ismail Khan) TBCC (Tuberculosis control centre).

This cross sectional study was aimed to investigate the impact of forced internal displacement on the adherence to the treatment protocols and treatment outcomes among internally displaced TB patients. Both IDPs (displaced TB patients) and NIDPs (settled area TB patients) diagnosed with pulmonary TB were included in the study that had been enrolled/registered in five TB control centres which are NWA, Bannu, Lakki Marwat, Dera Ismail Khan, Fata Region Bannu and Public-Private Mix TB clinics. Study participants were recruited on the basis of inclusion criteria, whom the treatment schedule was going to complete during the study period (January 2016- March 2017). All patients who had an incomplete address, unwilling to participate, younger than 18 or older than 65 years of age were also excluded from the study. TB patient’s record which was present in the TB center registers were assessed and then inclusion and exclusion criteria of the study were applied. A total of 703 patients form TB centers were assessed in this study, out of which 330 patients full filled the inclusion criteria and were assigned equally to both IDPs and NIDPs study groups (Fig.1).

### Data Collection Instrument:

In this study, structured questionnaire was used as a research instrument to obtain the data from the TB patients regarding adherence to TB treatment which included the number of prescribed doses of medicine per day and number of days with complete or partial missing of prescribed doses. VAS (visual analogue scale 10-100%) was used to calculate the proportion of dosages taken by the patient in the previous month.^12^ Patients were asked to report the number of anti-tuberculosis pills they took the day before the survey as well as two days, and four days before the survey. More than 25% of the pills missed in the last four days classified as non-adherent. If only 1-day dose was missed than classified as satisfactory and complete adherent if no pill was missed a pill in the last four days. The adherence measured by the VAS was classified as unsatisfactory (<80%, that is rating a value lower than 8 on the VAS), satisfactory (≥80% but less than 100%) or complete satisfactory (100%) adherence. The second part questioners administered to patients with the following sections; demographic information, socioeconomic status, quality living place, number of rooms/tents, room density, movement of family and access to the health centre.

### Ethical Considerations:

The bioethical committee of Quaid-i-Azam University, Islamabad approved the study on 18 May 2016 (BEC-FBS-QAU-68) and In-charge of TB control centres granted permission to conduct the study.

### Statistical Analysis:

The data was analyzed SPSS 21^®^ chi-square test was applied to assess association and ordinal logistic regression were used to estimate the impact of displacement related factors on adherence.

## RESULTS

The treatment outcomes of the registered participant are shown in (Table-I) and it was found that 39.4% were “cured”, 25.5% have completed treatment, 13.6% showed “default treatment” whereas 2.4% of TB patients showed “failure”. No patient was reported died among the registered participants of the study. The number of “cured & completed treatment” patients was less among IDPs (35.2% and 18.2%) as compared to NIDPs (43.6% and 25.5%) whereas more number of defaulted and failure cases were identified among IDPs (21.8% and 3%) as compared to NIDPs (13.6% and 2.4%).

**Table-I:** Comparison of the treatment outcomes and adherence among the TB of internally displaced patients and settled areas patients.

TB Treatment Outcomes	IDPs n (%)	NIDPs n (%)	Total n (%)	Chi-square
Cured	58 (35.2)	72(43.6)	130 (39.4)	0.101
Completed Treatment	30 (18.2)	54(32.7)	84 (25.5)	0.002
Defaulted Treatment	36 (21.8)	9(5.5(9)	45 (13.6)	0.000
Failure	5 (3.0)	3(1.8(3)	8 (2.4)	0.474
Not Documented	36 (21.8)	27(16.4)	63(19.1)	0.207
***Adherence***				
Adherent	65(39.4)	84 (50.9)	149 (45.1)	0.045
Partial Adherent	55 (33.3)	63 (38.2)	118 (35.8)	0.358
Non Adherent	45 (27.3)	18 (10.9)	63 (19.1)	0.000

IDPs (Internally displaced TB patients) NIDPs (Settled area TB patients).

The prevalence of the non-adherence measured by the VAS scale is shown in Table-I. The (27.3%) of IDPs were non-adherent to the TB treatment and NIDPs have only (10.9%) of such cases. Similarly, (33.3%) of IDPs and (38.2%) form NIDPs were found to be partially adherent to the medications. The adherence to TB treatment was more in NIDPs (50.9%) as compared to IDPs (39.4%) with p-value 0.045 vice versa non-adherent to the treatment was more among IDPs as compared to the NIDPs. There was no significant difference in partial adherence between IDP and NIDPs (33.3% in IDPs versus 38.2% in NIDPs, p 0.35). By adding all the above variables in (Table-II) which were mostly changed after displacement it was identified that the IDPs patients have significantly affected the adherence as compared to the NIDPs with (p=0.004). The estimated odds ratio is 0.223, which means that IDPs have 0.223 lesser probability to TB adherence is as compared to settled areas patients.

**Table-II:** Ordinal logistic regression analysis of adherence with displacement related factors (odd ratios and 95% confidence interval (n=330).

Variables	Odds Ratio	P-value	95% confidence interval
***Age (year)***			
15-25	Reference		
26-35	2.61	0.03	1.06 6.43
36-45	3.47	0.01	1.23 9.79
46-55	1.18	0.73	0.44 3.11
55-65	2.65	0.06	0.95 7.37
***Gender***			
Male	Reference		
Female	2.63	0.00	1.49 4.66
***Marital Status***			
Married	Reference		
Single	2.12	0.12	0.80 5.56
Widower	0.86	0.86	0.16 4.55
***Residency***			
Village	Reference		
City	0.97	0.93	0.53 1.76
Camp	0.13	0.00	0.05 0.36
***Education***			
No school	Reference		
Elementary	1.26	0.60	0.51 3.09
High school	3.62	0.01	1.33 9.90
College	4.18	0.01	1.37 12.07
Religious school	1.73	0.51	0.33 8.88
Higher education	0.41	0.50	0.03 5.49
***Number of rooms/tents***			
1room	Reference		
2-3 rooms	1.40	0.46	0.56 3.44
More than 4 rooms	1.22	0.69	0.43 3.49
1 tent	4.84	0.05	0.98 23.8
2-3 tents	4.05	0.02	1.21 13.5
More than 4 tents	0.57	0.80	0.07 43.8
***Access to Health centre***			
Not Easy Access	Reference		
Easy Access	2.03	0.06	0.96 4.30
Movement of Family Stable for > than 3 months	Reference		
Unstable < than 3 months	0.40	0.00	0.21 0.75
***Quality of place of living***			
Good	Reference	
Very good	1.42	0.35	0.67 2.99
Poor	1.88	0.15	0.79 4.48
Very poor	0.68	0.41	0.27 1.70
***Room density***			
Low density	Reference		
High density	1.16	0.67	0.56 2.39
Overcrowded	0.46	0.04	0.22 0.97
***Socio-Economics***			
Very poor	Reference		
Poor	2.34	0.01	1.15 4.76
Middle-income level	11.38	0.000	4.97 26.05
Rich	6.87	0.022	1.27 37.20
***Participants***			
NIDPs	Reference		
IDPs	0.223	0.00	0.09 0.54

IDPs (Internally displaced TB patients) NIDPs (Settled area TB patients).

## DISCUSSION

Tuberculosis is a major public health problem among refugees and internally displaced persons globally who have a higher prevalence of TB and more drug-resistant cases as compared to settled area populations.^8^ Families with poor socioeconomic conditions are often vulnerable to tuberculosis due to densely populated poor living conditions. TB patients from these groups adhere poorly to their treatment and show non-compliance to their treatment and produce drug resistance and transmission. The current study highlights, the assessment of the adherence to TB treatment amongst IDPs and NIDPs, which is one of the major indicators of successful anti-TB treatment outcomes. In the present study, the success rate of the treatment outcomes (cured+completed treatment) among IDPs was only (53.4%), which is markedly lower than the WHO recommendation i.e. ≥85%.^13^ Such low success of the treatment among IDPs not only compromise the treatment of that individual but also pose a significant threat to the community in terms of transmission, relapse and drug resistance.^14^-^16^ The treatment success rate among the participant of the study depends upon the nature of the care they received and adherence to the treatment protocols. In our study, the adherence was lowered among IDPs as compared to NIDPs having a similar condition of the patients as reported previously.^15^-^17^ As in our study, we have observed that fully adherent patients are only 39.4%, which showed a significant impact on the treatment outcomes of TB. Similarly, the present study showed compliance with the previously reported studies in displaced peoples of China, Afghanistan, Ethiopia and Syrian refugee camps in Jordan (Table-I).^18^-^21^

After investigating the association of adherence with gender, marital status there was no significant association between them, but the level of education was found a contributing factor towards adherence (Table-II). Similarly to other studies in which the education was found a contributing factor while the other gender and marital status were insignificant.^22^ The nature of residency has shown a significant effect on adherence and the patients from the IDPs living in the camps have shown non-adherence to the medication higher than patients living in village or cities. This finding is in consistence with previous reports.^17^,^18^,^23^

Our study specifies that non-adherence among IDPs TB patients is common therefore the TB treatment program, health care providers should assure collaboration between the local and external stakeholders that assure all IDPs TB patients understand the significance of adherence in management of TB.

### Limitation of the study:

Those TB patients who were not identified or who had no registration in study selected area TB control centers may have introduced a selection bias. Despite these limitations, the current study has numerous strong points; the study involved reasonably large numbers of IDPs and NIDPs, individuals that enabled a more close inspection of the influencing issues of adherence due to displacement.

## CONCLUSIONS

This study has made endeavors to give understanding into adherence to TB treatment and its determinants among IDPs TB patients of NWA. Overall the results of this study revealed a poor adherence to the TB treatment medications among IDPs as compared to local residents.

### Author’s Contributions:

**FUK:** Contributed to design and implementation of study as well as data collection, and manuscript writing. **AK:** Contributed in data collection and review of the draft. **ZA:** Contributed to study design and data analysis and review of the manuscript. **MKT & AUR:** Helped in data analysis and reviewed final draft. **TUR:** Conceptualized and implemented study design and helped in initial draft and review of the manuscript.
